# HarmonizR: blocking and singular feature data adjustment improve runtime efficiency and data preservation

**DOI:** 10.1186/s12859-025-06073-9

**Published:** 2025-02-11

**Authors:** Simon Schlumbohm, Julia E. Neumann, Philipp Neumann

**Affiliations:** 1 Chair for High Performance Computing, Helmut-Schmidt-University, University of the Federal Armed Forces Hamburg, Holstenhofweg 85, 22043 Hamburg, Hamburg Germany; 2 Center for Molecular Neurobiology Hamburg (ZMNH), University Medical Center Hamburg-Eppendorf (UKE), Falkenried 94, 20251 Hamburg, Hamburg Germany; 3 Institute of Neuropathology, University Medical Center Hamburg-Eppendorf (UKE), Martinistraße 52, 20251 Hamburg, Hamburg Germany; 4 IT-Department, German Electron Synchrotron (DESY), Notkestraße 85, 22607 Hamburg, Hamburg Germany; 5 High Performance Computing and Data Science, University of Hamburg, Notkestraße 85, 22607 Hamburg, Hamburg Germany

**Keywords:** Batch effects, Proteomics, Computational efficiency, Big data, Dataset integration

## Abstract

**Background:**

Data adjustment is an essential tool for increasing statistical power during analysis, for example in case of complex multi-experiment data from (single-cell) RNA, proteomics and other omics data. Despite its benefits, data integration introduces internal biases—so-called batch effects. Due to the inherent presence of missing values by such methods and their additional introduction by means of data integration, renowned algorithms such as ComBat and limma are unable to perform batch effect adjustment. Recently, the HarmonizR framework was presented for these cases, which is a tool for missing value tolerant data adjustment.

**Results:**

In this contribution, we provide significant improvements to the HarmonizR approach. A novel blocking strategy is introduced to severely reduce runtime, while still supporting parallel architectures. Additionally, a “unique removal” strategy has been integrated into HarmonizR to maintain even more features for adjustment in datasets, showing a feature rescue of up to 103.9% for our tested datasets. In this work, we show (1) severely improved runtime for both small and large, real datasets and (2) the ability retain more features from the integrated dataset during adjustment, showing a feature rescue of up to 103.9% for our tested datasets.

**Conclusion:**

The proposed improvements tackle the previous shortcomings of the published HarmonizR version. Since HarmonizR was mainly developed for dataset integration on rare tumor entities, it did not include runtime improvements beyond parallelization, which has been addressed in this update. An additionally welcome update regarding improved feature rescue furthermore enhances the algorithms ability to quickly and robustly perform batch effect reduction.

## Background

### General

Omics data, such as genomics, transcriptomics, proteomics and metabolomics are often used to investigate molecular changes in health and disease [1]. Yet, individual cohort measurements may often lack statistical power resulting from laboratory pipeline or measurement technique limitations, which is usually compensated by data integration to increase cohort size. Among this data, be it from proteomics, single-cell-RNA sequencing and the upcoming field of single-cell proteomics, high data incompleteness is reported [2, 3].

Dataset integration commonly leads to the introduction of an internal bias—the batch effect—coming from for example different measurement techniques used for every individual chunk of data to be appended [4]. To reduce this effect and unveil suppressed biological meaning, batch effect adjustment algorithms are of need. Based on the given field of study, batch effect reduction is already well established, especially in fields which show a small internal bias and high data completeness like DNA-methylation or the transcriptome, yielding easily comparable data [5–7]. This, however, is not the case for fields such as proteomics. As described by Aslam et al., the proteome is highly dynamic and suffers from high experimental variance [8]. In 2003, the tandem mass tag (TMT) quantification technique was found to reduce the data incompleteness of measured proteomic data, yet, without removing the problem entirely [9]. Additionally, TMT measurements are based on TMT plexes, which by themselves introduce a batch effect between the plex measurements within the dataset. A positive correlation between the amount of plexes—synonymously called batches—and the amount of introduced missing values in the resulting measurements has also been described [10].

Established algorithms for batch effect adjustment exist and are commonly used, namely ComBat and limma [4, 11], which however have not been developed to deal with missing data, making direct evaluation of their batch effect reduction on incomplete dada impossible. The recently developed HarmonizR framework resolved this issue, while still relying on the two aforementioned batch effect adjustment methods. We focus on the added-value of new HarmonizR features in the following, with a focus on runtime improvements. For detailed qualitative and quantitative agreements of the HarmonizR approach, the reader is referred to our previous publication [12]. To our knowledge, HarmonizR is the only existing data integration tool to date that allows for arbitrarily incomplete omic data to be considered and is hence used for comparison against the advancements made within in this work. Furthermore, HarmonizR has been shown to outperform imputation—as shown in its original publication—and batch effect correction using internal reference samples [13].

Furthermore, Jaramillo-Jiminez et al. showed in their recently published 2024 paper the effectiveness of ComBat-based approaches for harmonization and denotes HarmonizR as particularly effective, underlining the validity of the approach [14].

### Motivation

While it has been clearly demonstrated that HarmonizR reduces batch effects very effectively, its computational efficiency and the handling of certain, currently unadjustable features in an integrated dataset require further consideration. This work aims at tackling these issues, forging a robust algorithm able to handle issues far beyond the original HarmonizR framework.

#### Computational performance and sub-matrix growth

**Fig. 1:**
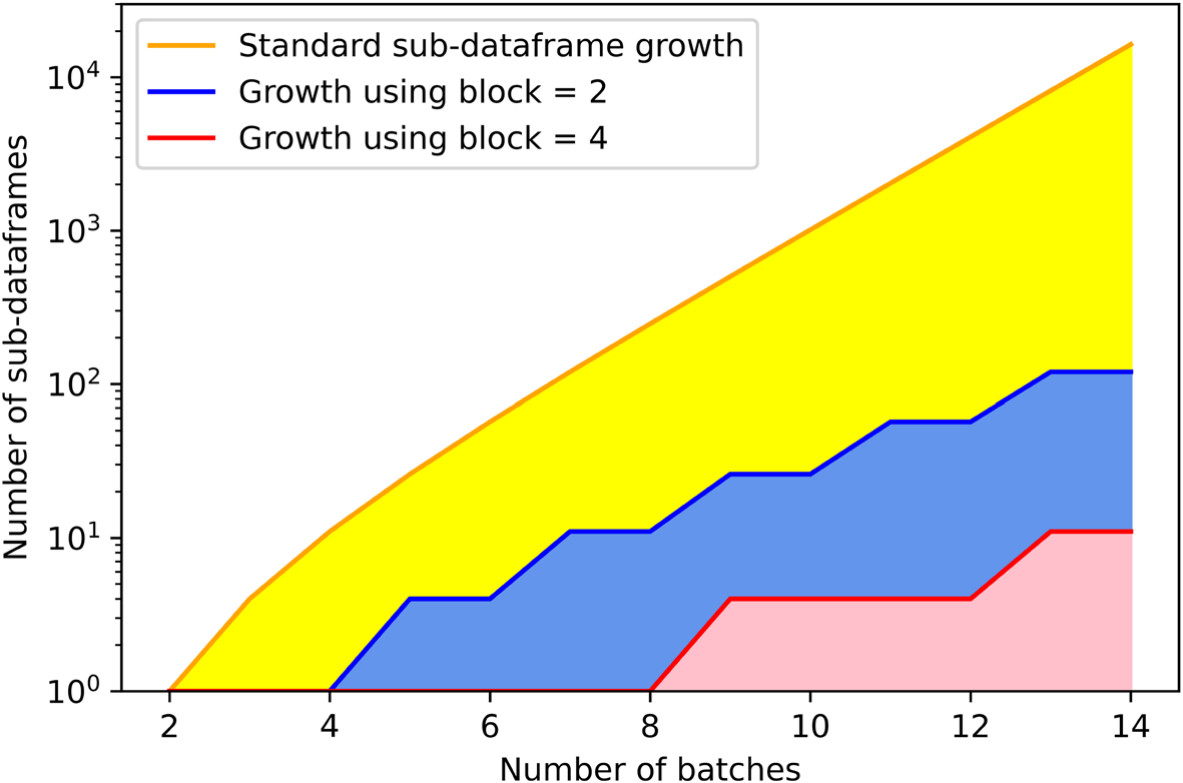
Potential sub-matrix growth shown for up to 14 batches. The y-axis is in a logarithmic scale. The orange curve shows the maximum expected growth using default HarmonizR according to the sum of binomial coefficients $t_{n,\text{max}} = \text{min} \left( { F}, \sum _{k=2}^{n} {n\atopwithdelims ()k} \right)$ with the maximum number of features *F* and total number of batches *n* [12]. The blue and red curves show the growth when using the blocking approach presented in this paper with the parameters 2 and 4 respectively

HarmonizR is based on a matrix dissection approach applied to the integrated dataset. This allows to call the desired, underlying batch effect adjustment algorithm (limma, ComBat) on the created sub-matrices, adjust the sub-matrices independently from each other and execute this adjustment simultaneously by leveraging multiple compute cores or compute nodes of a cluster, and re-integrate the results afterwards [12]. Yet, this matrix dissection easily produces a big number of sub-matrices, which grows exponentially with an increasing number of batches as shown in Fig. 1.

A feature (protein/gene/etc.) is sorted into a sub-matrix based on the combination of batches for which it has sufficient numerical values in. For the underlying algorithms (ComBat/limma), sufficient numerical values is defined as at least two numerical values within a batch for the given feature. This means that if a feature has one a singular or none numerical values in a batch, that batch is considered absent or “missing” for that feature. A feature’s combination is then defined by all batches it has sufficient numerical values in (e.g. if a features has sufficient numerical values in batch 1, 2 and 4 but not 3, its combination will be {1, 2, 4}).

If a sub-matrix holding features with this very combination does not exist yet, it is created from scratch and the feature is added. Naturally, the more batches there are in a given dataset, the more possible combinations can occur. This directly influences computational efficiency since increasing amounts of sub-matrices have a negative impact on the program’s runtime.

#### Inability to adjust matrices with singular features

Another problem with the current implementation of the HarmonizR framework is the inability to adjust a single feature by itself. This means that whenever a feature ends up alone in a sub-matrix at the end of the dissection step, the matrix cannot be adjusted and needs to be discarded in this step, resulting in data loss.

A feature ends up by itself whenever the combination of batches it appears in/has sufficient numerical values in is unique to that feature. The impact of this problem is very dataset-dependent.

## Methods

### Sorting and blocking for increased efficiency

**Fig. 2:**
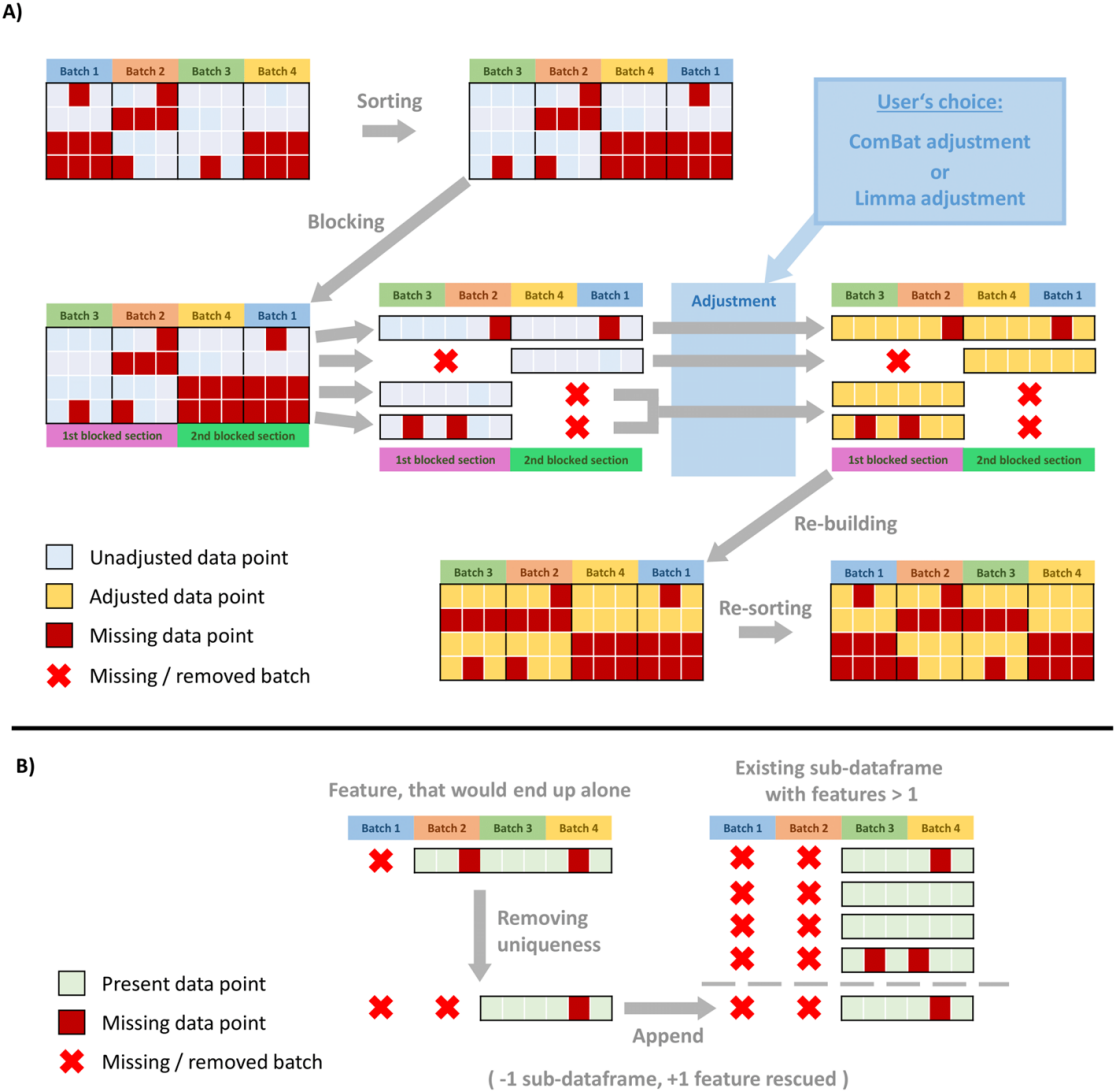
Overview of the major features of the updated HarmonizR version. **A** Sorting, blocking and the respective dissection are exemplarily shown in the flowchart. The adjustment is then performed on the newly created sub-matrices. The matrix gets rebuilt and re-sorted to match the initial order of the input data. The sorting (plus re-sorting) and the blocking are new features of this software version. The shown dissection has been a feature present in the previous version. **B** The mechanism behind the removal of unique, standalone features is also exemplarily shown. Once a unique combination is found it is cropped such that it matches at least one other feature in the dataset ensuring the rescue of the feature. It is opted for removing as little information as possible by cropping as few batches as possible. The removal of unique combinations is not influenced by prior or subsequent sorting and takes place before blocking. Sorting, blocking and unique removal are completely optional features

Reducing the amount of batches significantly lowers the amount of created sub-matrices and thus increases algorithmic performance. We implement this through a novel blocking parameter, which blocks neighboring batches together and treats them as one during the matrix dissection. Setting the parameter to 2 for example will attempt to always block two neighboring batches together and treat them as one during the splitting phase. Note that blocking does not introduce the assumption that the neighboring batches are biologically similar nor will the batch effect be ignored within blocked sections upon adjustment. It is only carried out to technically reduce the number of sub-matrices. The batch effect reduction itself will still be carried out on the blocked batches separately by the respective algorithm (ComBat/limma) on the finest granularity based on the actual batch description. This effectively means that blocking has an effect on the matrix dissection, thus tweaking algorithm runtime and data completeness but has no effect on the behavior of ComBat and limma adjusting the batch effect for all present batches. As shown in Fig. 1, blocking drastically decreases the amount of sub-matrices passed on towards the adjustment phase. In case of a remainder, the remaining batches are considered separately. Figure 2a schematically shows how the blocking approach has been incorporated into the existing HarmonizR framework.

Using this approach, without exception, all batches that get blocked together need to satisfy the required data presence for the underlying adjustment algorithms: if one of the blocked batches does not hold enough numerical values for an adjustment of a given feature, all other batches blocked with that batch cannot be considered either for that same feature, hence leading to data loss.

As seen in Fig. 2a, neighboring batches are by default blocked together. This is adjustable by using the optional sorting parameter. The goal of sorting the batches in advance is to minimize data loss introduced by the blocking approach. Upon usage, the data gets rearranged based on one of several sorting approaches available. For instance, the batches can be sorted by their completeness. Here, completeness is being defined by the amount of missing values found within a batch. This approach is in the following called sparsity sort and can primarily prevent very complete batches to be discarded for many features since they will no longer be blocked together with very incomplete batches.

Alternatively, a Jaccard-index based sorting can be applied, which sorts batches based on where missing data is found within them and calculating similarities [15]. The Jaccard-index is a statistic for the similarity of two sets. In the context of this work, sets are the given batches. The more similar two batches, the closer they become in terms of vicinity within the data matrix when employing this sorting method. Furthermore, a seriation based sort can be applied, which is directly provided by Hahsler et al. in the R-based “seriation” package. Here, seriation sort reorganizes batches by evaluating their pairwise similarities, arranging them in an order that reduces disorder, thus uncovering underlying patterns or structures in the data. [16].

### Removal of unique combinations to increase number of rescued features

The underlying algorithms of the HarmonizR framework are limited in their ability to properly handle input matrices with only a singular feature present, which is the direct result of a unique batch combination. Instead of discarding these features, a new functionality has been developed, attempting to even rescue these features. The goal is to get rid of unique combinations during dissection while simultaneously retaining as much measured data as possible. A schematic, exemplary depiction is provided in Fig. 2b. The essence of the approach is to crop a given feature in a way that its newly cropped batch combination fits at least one other feature within the dataset, effectively removing the given feature’s uniqueness. In the end, the feature can be considered with minor data loss. As a positive side effect, this procedure already reduces the amount of sub-matrices by one for every found, unique feature.

This new functionality can proof vital for many datasets across multiple omics types such as for example single-cell-RNA-seq data (which can contain a huge amount of zeros due to limitations in the data generation process [2]) or other large, non-TMT proteomic datasets alike. Both singular feature adjustment and blocking can be combined to increase computational performance and maximize data rescue at the same time.

## Results

Tests regarding computational performance and adjustment quality have been conducted on three TMT datasets [17–19], (17, 23, 42 batches, respectively). Additionally an artificial dataset and a single-cell-RNA seq dataset [20] have been evaluated.

### Blocking: computational performance

**Fig. 3:**
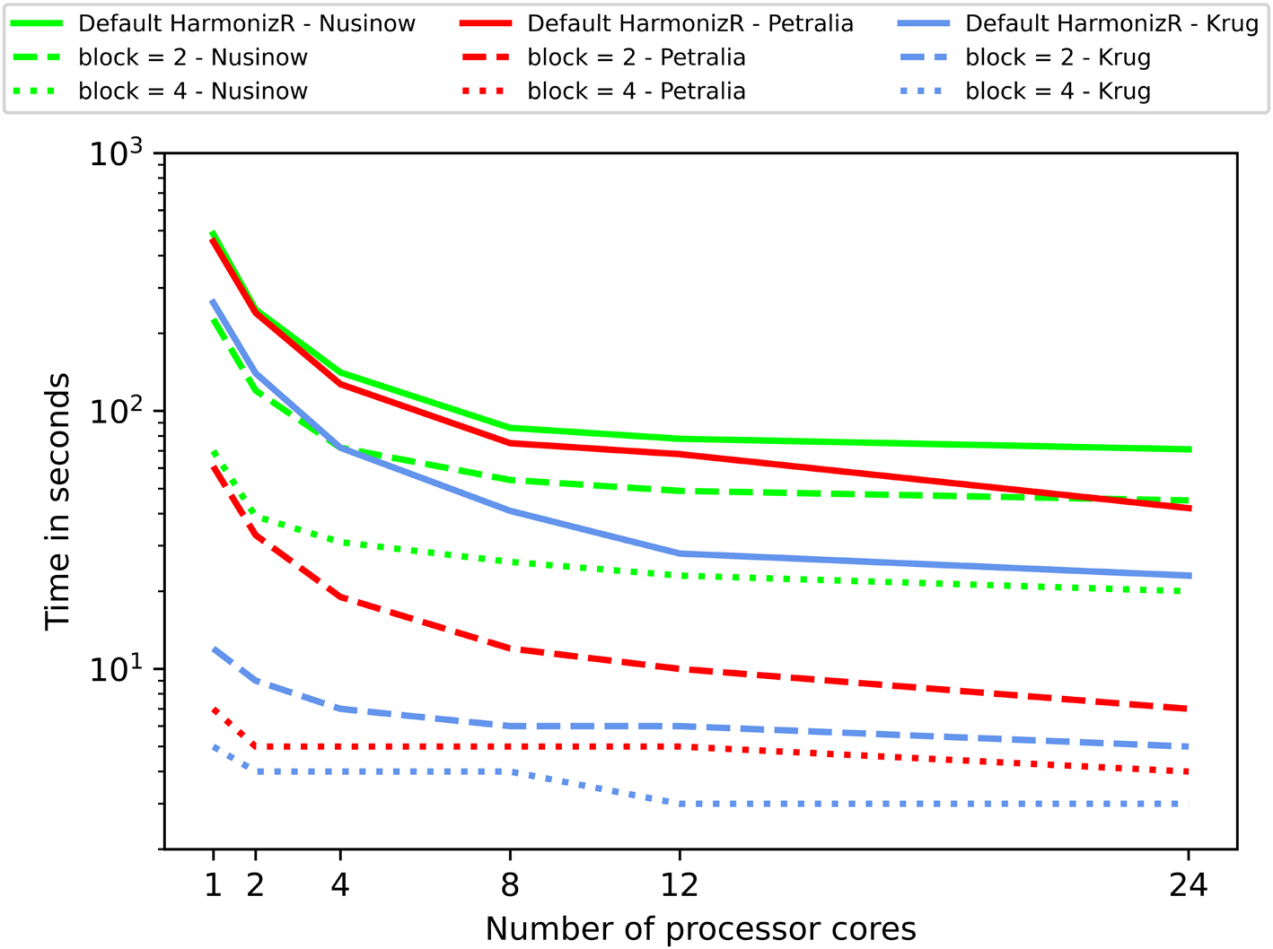
The runtime in seconds (y-axis) of the adjustment step of HarmonizR. Tests have been carried out on three datasets using 1, 2, 4, 8, 12, and 24 processor cores. These steps are depicted on the x-axis. Computed on an Intel Xeon Gold 6226, 2.70 GHz, 2 × 12 compute cores, 96 GB RAM

**Fig. 4:**
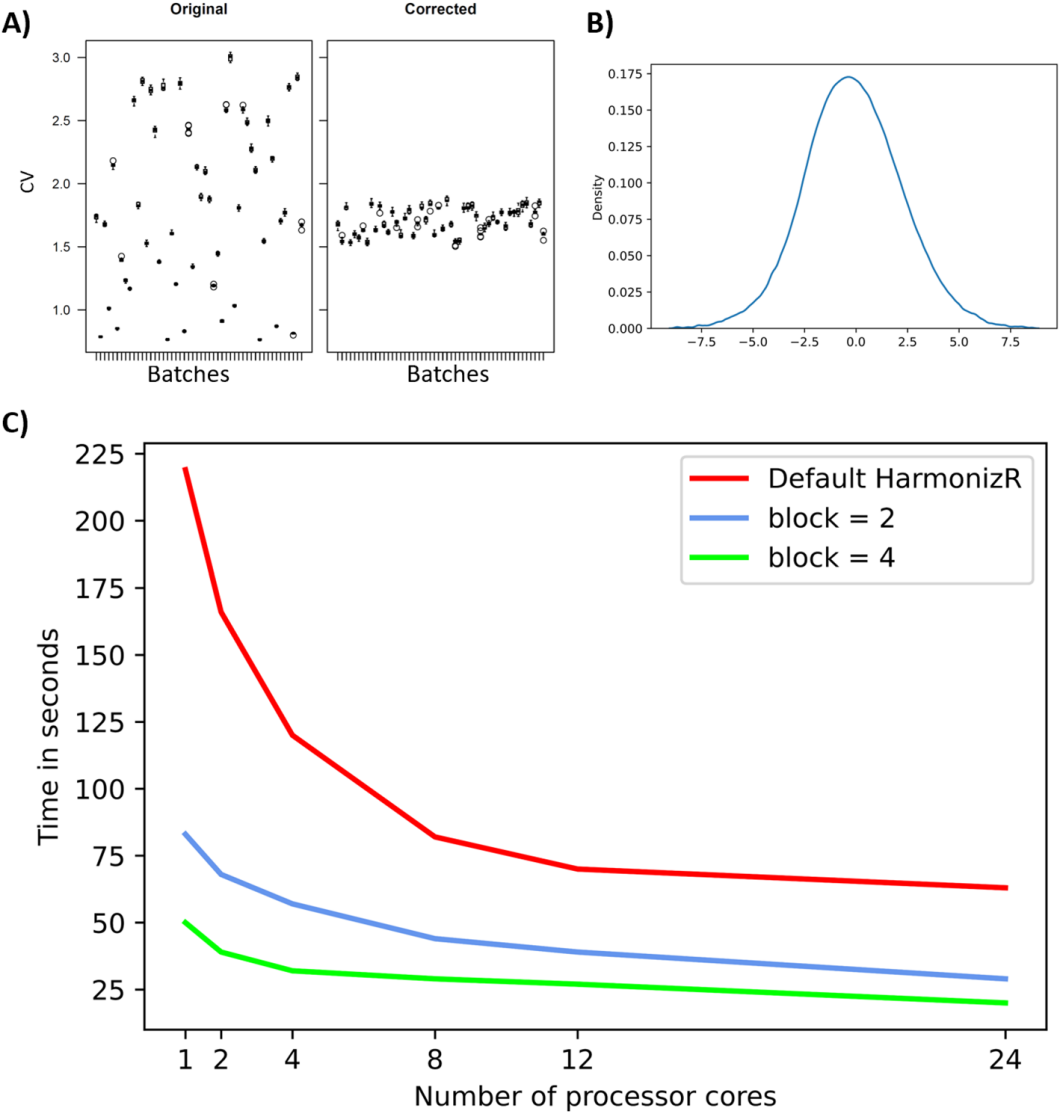
Tests for HarmonizR on an artificially created dataset. It contains 50 batches in total with 10 samples each as well as 15,000 features to be accounted for. Values within each batch are normally distributed and have values missing-not-at-random. **A** Coefficient of variation (y-axis) shown for both the artificial dataset prior to adjustment (left) and after HarmonizR adjustment (right), showing both the artificially introduced batch effect as well as its reduction. **B** Density plot for all values within the dataset prior to the adjustment. Since all batches have been created based on a normal distribution plus an additive and multiplicative factor, an overall normally distributed dataset is to be expected. **C** The runtime in seconds (y-axis) of the HarmonizR adjustment part. Here, runs have been performed with no blocking and the block parameter set to 2 and to 4, respectively. Tests have been carried out using 1, 2, 4, 8, 12, and 24 processor cores. These steps are depicted on the x-axis. Computed on an Intel Xeon Gold 6226, 2.70 GHz, 2 × 12 compute cores, 96 GB RAM

To a large extent, the implementation of the blocking functionality is justified by the overall runtime improvement of the HarmonizR framework. As described in the previous paper by Voß and Schlumbohm et al. in 2022, the major part of the algorithm is executed in parallel. While this greatly improved performance on big datasets, proper exploitation of the structure of the input matrix can further improve runtime with the described blocking approach. HarmonizR runtime using blocking is compared to the runtime of the default (parallelized) HarmonizR adjustment, see Fig. 3 and Table 1 for the three TMT datasets and Fig. 4 for an artificial dataset. Beyond the artificial data shown in Fig. 4, an even larger study has been performed, which simulate a dataset larger than the ones shown here and the measured runtime can be viewed in Online Appendix Figure 1.

**Table 1:** Factors at which parallel runtime was sped up using blocking. Measurements for all three datasets are included and the factor is calculated from the measured runtime of a default HarmonizR run divided by the measured runtimes of runs including blocking with block = 2 and block = 4, respectively (the latter are also shown in seconds). Computed on an Intel Xeon Gold 6226, 2.70 GHz, 2 × 12 compute cores, 96 GB RAM

	Krug et al. 2020	Petralia et al. 2020	Nusinow et al. 2020
Ratio: Default to block = 2 (1 core/sequential)	Runtime: 12 s Factor: 21.92	Runtime: 61 s Factor: 7.44	Runtime: 277 s Factor: 2.14
Ratio: Default to block = 2 (4 cores)	Runtime: 7 s Factor: 10.29	Runtime: 19 s Factor: 6.68	Runtime: 72 s Factor: 1.96
Ratio: Default to block = 2 (12 cores)	Runtime: 6 s Factor: 4.67	Runtime: 10 s Factor: 6.80	Runtime: 49 s Factor: 1.59
Ratio: Default to block = 4 (1 core/sequential)	Runtime: 5 s Factor: 52.60	Runtime: 7 s Factor: 64.86	Runtime: 70 s Factor: 6.93
Ratio: Default to block = 4 (4 cores)	Runtime: 4 s Factor: 18.00	Runtime: 5 s Factor: 25.40	Runtime: 31 s Factor: 4.55
Ratio: Default to block = 4 (12 cores)	Runtime: 3 s Factor: 9.34	Runtime: 5 s Factor: 13.60	Runtime: 23 s Factor: 3.39

The three real datasets consist of proteomic TMT measurements: a breast cancer dataset from Krug et al. [17], a brain cancer dataset from Petralia et al. [18] and various cancer cell lines from diverse lineages combined in a dataset from Nusinow et al. [19].

For all datasets, a block parameter of two significantly reduced the runtime for these datasets. Blocking even more batches resulted in a reduction of time-to-solution by an order of magnitude (or even more) for the data adjustment. The new approach reduced runtime in these cases by factors of 1.59 to 64.86 (see Table 1 for more details).

When scaling up the number of used processor cores, a decrease in runtime could be observed for both the default HarmonizR and the blocking variants, respectively. A saturation of the speedup was observed between 4 and 12 cores. The three introduced sorting approaches naturally take time to be executed. Based on the chosen sorting method and the given dataset, these times will vary slightly. The measurement results are shown in the Table 2.

**Table 2:** Time for sorting in seconds for all three introduced sorting methods: sparsity-based sorting, seriation-based sorting implemented by Hahsler et al. [16] and Jaccard-based sorting using the Jaccard-index [15]. Measurements for the three TMT datasets. Computed sequentially on an Intel Xeon Gold 6226, 2.70 GHz, 2 × 12 compute cores, 96 GB RAM

	Krug et al. 2020	Petralia et al. 2020	Nusinow et al. 2020
Sparsity-based	< 1 s	< 1 s	< 1 s
Seriation-based	21 s	23 s	58 s
Jaccard-based	26 s	28 s	86 s

As can be derived from the table, sparsity-based sorting finishes almost instantly and therefore does not come with a significant runtime overhead. The more sophisticated seriation-based sorting and Jaccard-based sorting take longer times but generally improving feature rescue, which will be shown in the upcoming section.

Optimal HarmonizR parameters (blocking and sorting) are dataset-dependent. For the datasets explored in this work, a block size of 2 proved to be a solid balance in terms of adjustment quality and runtime. For the same data, Jaccard-based sorting proved most effective most of the time.

Furthermore, memory requirements of HarmonizR with respect to the three TMT datasets has been explored and measured. The results are shown in Table 3. Total allocated memory denotes the sum of all memory allocated at any point during the execution of the algorithm. This includes allocated, used and subsequently deallocated memory throughout a run. Peak random access memory (RAM) usage describes the point in time during which the most memory has been allocated at once, representing the maximum memory footprint.

**Table 3:** Total allocated memory and peak RAM usage (in megabyte) of the HarmonizR algorithm when executed with respect to the three differently sized datasets from Krug et al., Petralia et al., and Nusinow et al., respectively

Dataset	Dimensions (samples x features)	Total allocated memory (MB)	Peak RAM usage (MB)
Krug et al.	153 x 13793	4010.5	196.7
Petralia et al.	249 x 9155	7885.4	191.0
Nusinow et al.	420 x 12970	29500.2	204.7

### Effectiveness of batch effect reduction

Increasing computational efficiency at an algorithmic level often goes hand in hand with a decrease in computational accuracy, which especially holds for algorithms surpassing natural computational complexity barriers, such as heuristic algorithms [21], iterative solvers [22] or the fast multipole method [23]. In the context of the HarmonizR framework, this would be due to a bigger loss in data points during the blocking approach. Quality control has been conducted with various metrics to confirm accurate batch effect reduction of the blocking version of HarmonizR. In the following, we demonstrate at practical examples, that our optimization approaches only have marginal impacts on the overall batch effect reduction compared to the original HarmonizR algorithm.

**Fig. 5:**
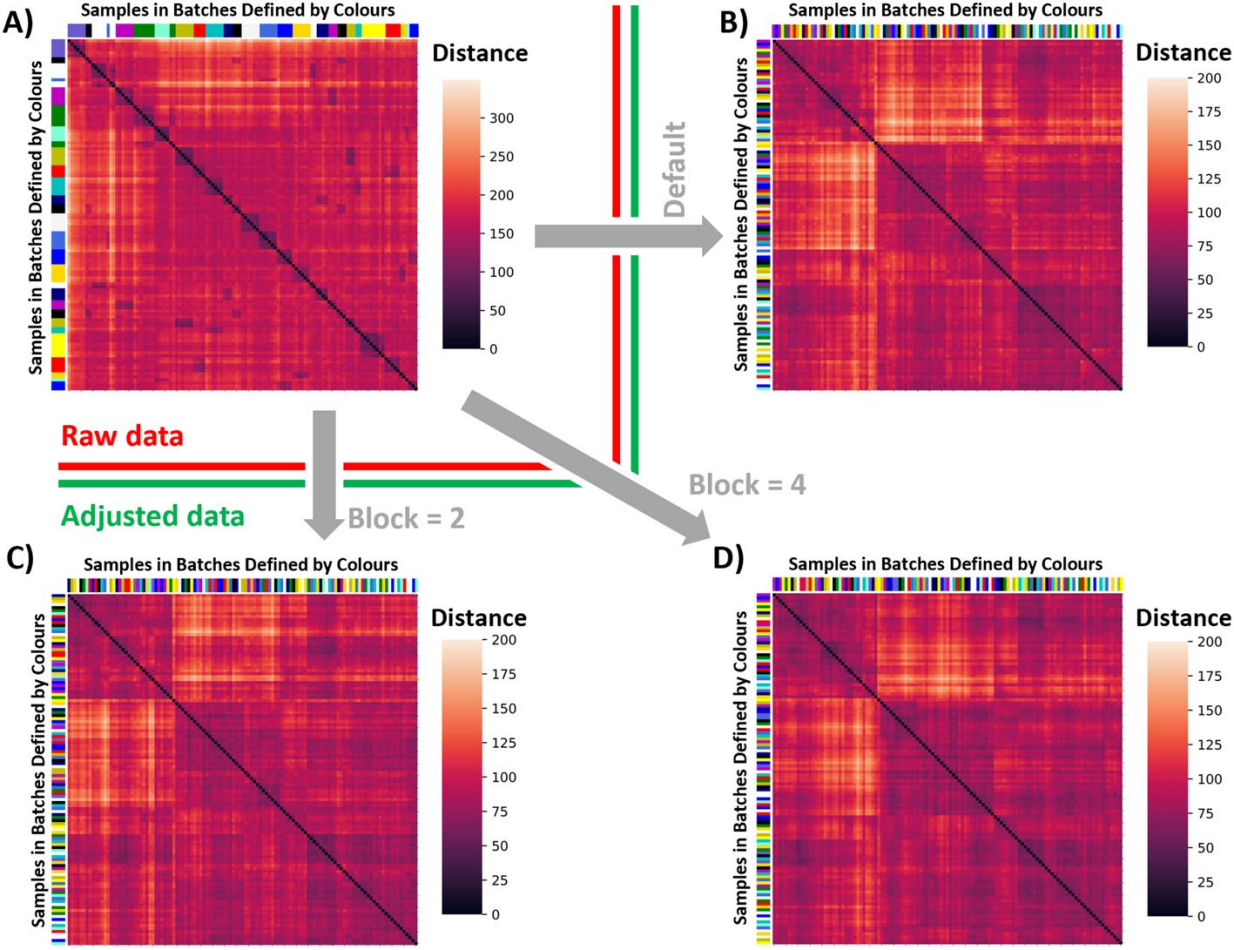
Heatmap representation of the distance matrices calculated from the dataset from Krug et al. (all samples vs. all samples); raw, adjusted by the default HarmonizR and lastly adjusted using the blocking approach. Next to the heatmaps, the respective legend is shown, defining the meaning of the shading. Color labels for samples indicate assignment to a certain batch. All adjustments were done using ComBat and without prior sorting. **A** The raw data. The batches are seen color-coded next to the heatmap. **B** The adjusted data from the default HarmonizR version without blocking. **C** The data arising from the blocking approach with the block parameter equal to 2. **D** The data arising from the blocking approach with the block parameter equal to 4

Figure 5 depicts a heatmap based on a distance matrix of all samples against all samples from the given dataset from Krug et al. [17]. For the raw, unadjusted data, there is a visible correlation of batches with themselves. This is apparent based on the block structures along the diagonal of the heatmap. These blocks can be matched with the batches, which are color-coded next to the heatmap in the first panel, showing the batch effect. The adjusted data does no longer show these structures along the diagonal. Furthermore, the adjusted distance matrices are more homogeneous than the raw data, which is what is to be expected after the batch effect adjustment (as can be seen from the respective color legend). The blocking approach yields proper adjustment of the data as well.

When blocking further (i.e. blocking the dataset from Krug et al. down to five batches total using the blocking parameter = 4), the quality in the adjusted features remains comparable.

**Fig. 6:**
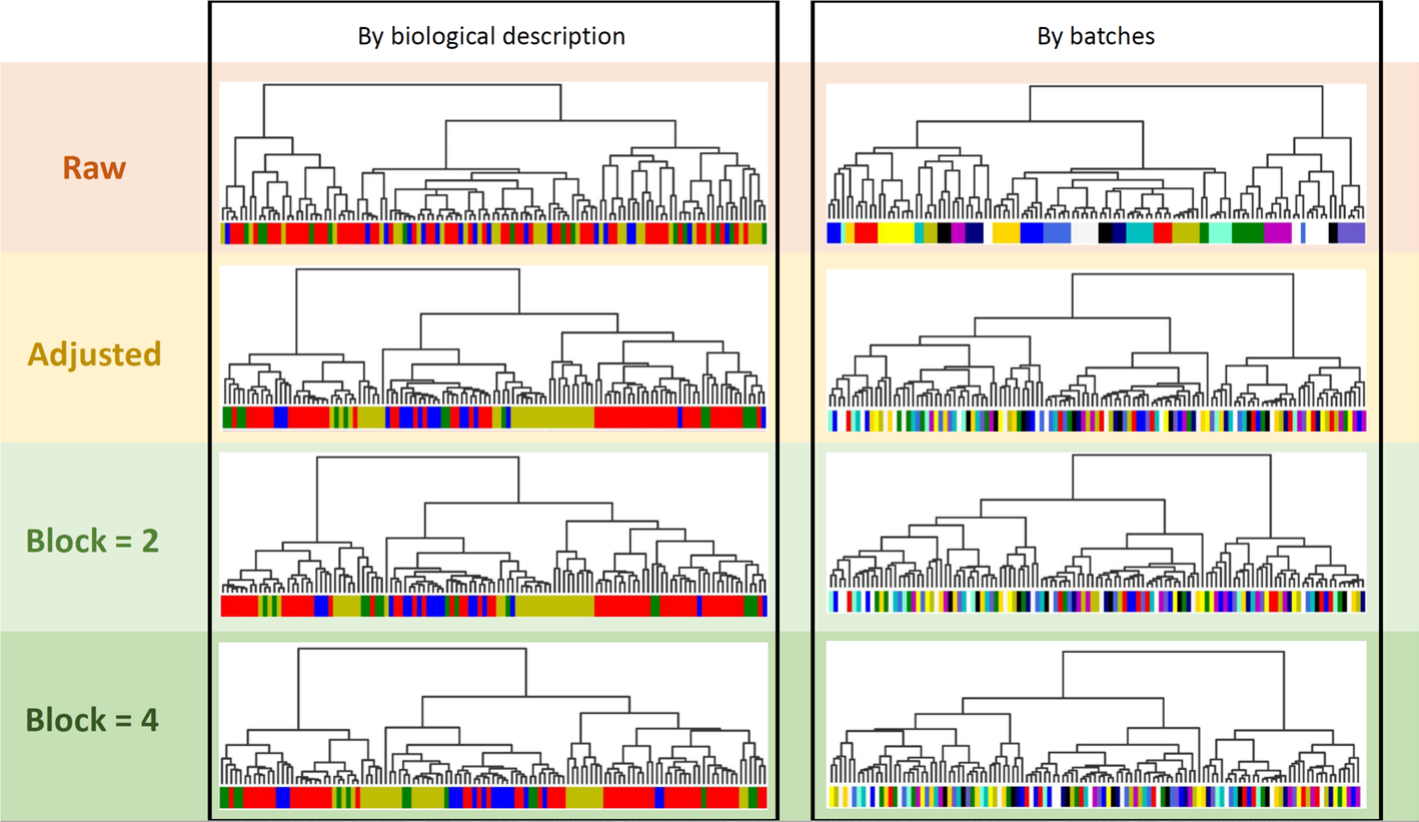
Dendrogram representation of clustering by batches and by biological description. This is done for Krug et al. with the raw dataset, the adjusted dataset (using default HarmonizR) and the adjusted dataset under the usage of blocking. block = 2 means always blocking 2 batches together if possible. block = 4 means always blocking 4 batches together, respectively. Leaves of the same color indicate the same affiliation of these samples in terms of either biology (left) or the batch they were measured in (right). All adjustments were done using ComBat as sub-matrix batch effect adjustment method without prior sorting

Figure 6 shows a dendrogram for the same data, for which clustering has been performed.

Within the raw data, the batch effect can be observed very well, given the clustering based on the 17 batches of the dataset (right) and not at all based on the biological description of the samples (left). The (by default HarmonizR) adjusted data is shaded in yellow and shows a proper batch effect reduction. Taking the blocking approach with a blocking parameter of 2 or 4 is highlighted in lighter and darker green. This approach still yields good results as can be derived from similar clustering results compared to the established, default HarmonizR adjustment since a clustering based on batches can no longer be observed for both blocking parameters and instead a clustering based on biological description is observable.

An additional test regarding effective batch effect reduction on single-cell-RNA seq data is shown in Online Appendix Figure 2 based on the dataset from Xin et al. [20]. This demonstrates the applicability of HarmonizR to single-cell sequencing data.

**Fig. 7:**
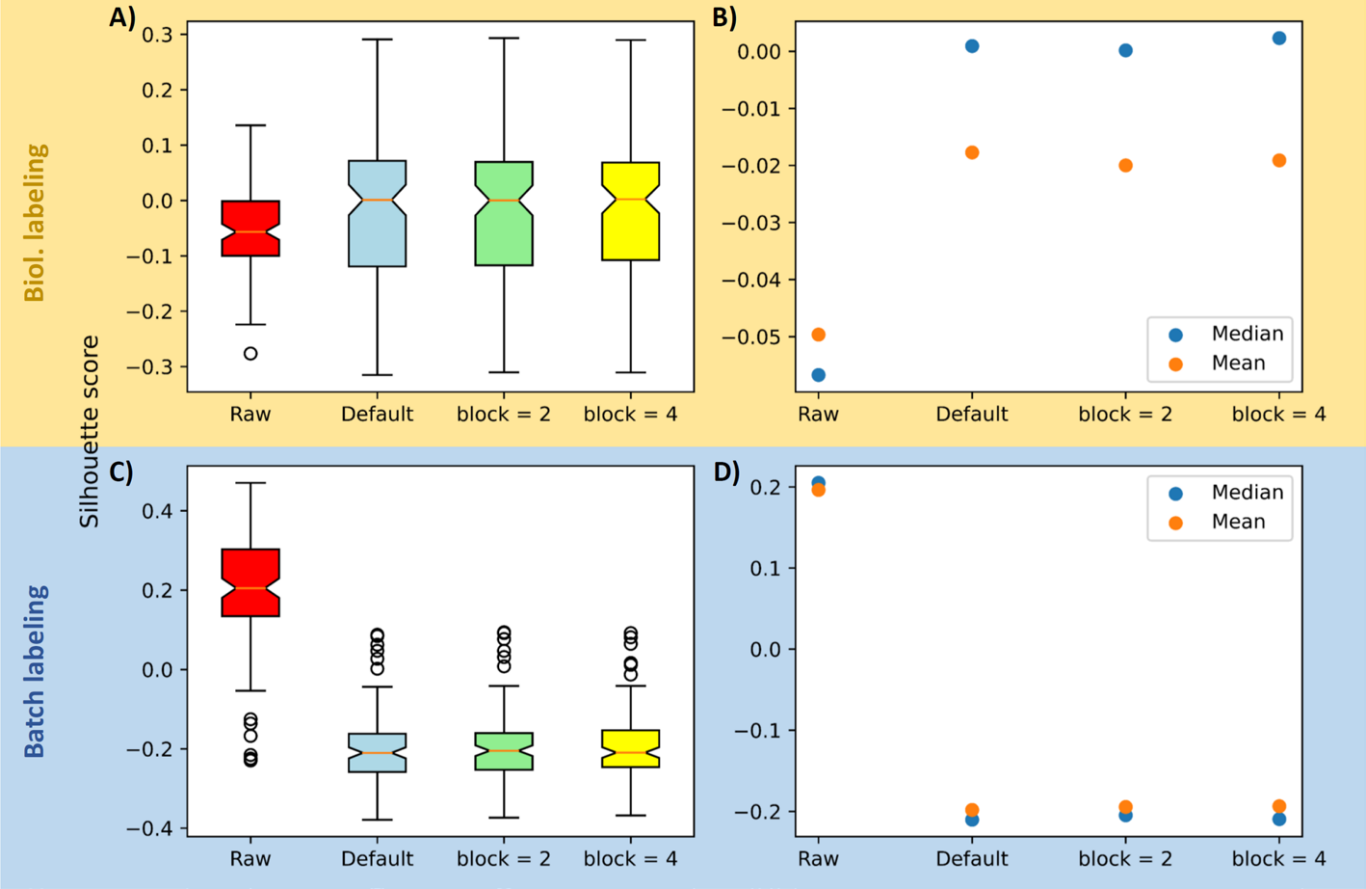
Silhouette score calculated for the dataset from Krug et al. The given granularity is shown on the x-axis. The silhouette score, shown on the y-axis, lies between − 1 and 1. 0 means randomly distributed, 1 means perfect clusters (all data points on top of each other within a cluster). − 1 describes the opposite of 1. All adjustments were done using ComBat in its default mode as sub-matrix batch effect adjustment method without prior sorting. **A** Boxplot depiction of the silhouette score using biological labeling. **B** The median (blue) and the mean (orange) of the boxplots directly to the left (biol. labeling). **C** Boxplot depiction of the silhouette score using labeling by batches. **D** The median (blue) and the mean (orange) of the boxplots directly to the left (batch labeling). In boxplots, 50% of the data points are inside the box (Q1 (Quartile 1) being the lower bound of the box (25%), Q3 being the upper bound of the box (75%)). Whiskers show all values beyond the box without outliers. Outliers were defined as Q3 + 1.5 * IQR (Interquartile range) (upper outlier) and Q1−1.5 * IQR (lower outlier). IQR being Q1–Q3

The silhouette score given in Fig. 7 for the dataset from Krug et al. underpins and further quantifies this statement.

The silhouette score ranges from − 1 to 1 and gives an estimate for how well the data clusters based on the given sample labels, which in this case were the biological descriptions for the top two plots of the figure and the batch description for the bottom two. 0 indicates a random clustering while 1 describes a perfect overlap of all points (based on their Euclidean distances) [24]. In the top two plots of the figure it is shown, that the mean and the median of the unadjusted data are lower than for its adjusted counterparts. The relatively small improvement is probably due to the curse of high dimensionality as described by Aggarwal et al. in 2001, who explicitly mention the commonly used $L_{k}$ norm as a culprit [25].

According to the authors, higher norm parameters do provide a poorer contrast between furthest and nearest neighbor. The here shown silhouette score is based on a missing value tolerant, Euclidean distance matrix and therefore falls under the $L_{2}$ norm.

Within the lower plots it is shown that the raw data tends to cluster based on batch description, which is not desirable and unlike what is seen for the adjusted data. The difference between raw and adjusted data can be seen nicely with the raw data having both mean and median at around 0.2 while the adjusted data shows values around − 0.2. This trend holds for basically all our experiments, including the blocking approach.

The same metrics were applied to the dataset from Petralia et al. and are shown in Online Appendix Figure 3, 4 and 5 respectively.

To show robustness regarding high amounts of missing values, their amount has been artificially increased from 23.73 to 50.39% exemplarily for the dataset from Krug et al.. The same quality metrics were applied and a reduced version of the results for this experiment is shown in Online Appendix Figure 6.

We further show the misclassification error has been calculated using a KNN classifier and is shown in Table 4 for the datasets from Krug et al. and Petralia et al..

**Table 4:** Comparison of the misclassification error between the raw datasets, the adjusted data using the default HarmonizR adjustment and two blocking approaches using a block size of 2 and 4 respectively. The tests were done on the datasets from Petralia et al. and Krug et al., using the KNN classifier provided by the Python package ’sklearn’. No prior sorting was performed

Approach	Raw data (%)	Default adjustment (%)	block = 2 (%)	block = 4 (%)
Misclassification error (Krug et al.)	57.2	27.4	27.0	26.5
Misclassification error (Petralia et al.)	71.3	19.4	19.6	20.2

We here restricted views to data from Krug et al. and Petralia et al. since there are proper biological labels to work with and compare against. Considering the dataset from Krug et al., a misclassification error can be shown to be 27.4% for the normal adjustment (no blocking), 27.0% for block = 2 and 26.5% for block = 4 without any hyperparameter optimization. The unadjusted data shows a misclassification error of 57.2% since clusters are mostly found within the same batch only. Considering the dataset from Petralia et al., a misclassification error can be shown to be 19.4% for the normal adjustment (no blocking), 19.6% for block = 2 and 20.2% for block = 4 without hyperparameter optimization. Unadjusted data here shows a misclassification error of 71.3%.

Upcoming, the impact of the removal of unique features has been evaluated (Table 5).

**Table 5:** The impact of the newly implemented removal of unique combinations and the resulting increase in rescued features/proteins in the output matrix (without prior sorting). The old and new HarmonizR versions are shown as well as raw ComBat and limma. The protein increase is shown in absolute numbers and percentage-wise. No blocking (and therefore no sorting) has been applied during the runs shown, which achieved maximal data rescue

Dataset	Raw ComBat/limma	Proteins in the output with the original HarmonizR version (considered proteins /total proteins)	Proteins in the output with the updated HarmonizR version: removal of unique combinations (considered proteins /total proteins)	Protein increase between old and new version
Krug et al. 2020	6744/13,793 (48.9%)	10,808/13,793 (78.4%)	13,740/13,793 (99.6%)	2932 (27.1%)
Petralia et al. 2020	3749/9155 (40.9%)	5216/9155 (57.0%)	9154/9155 (99.9%)	3938 (75.5%)
Nusinow et al. 2020	5098/12970 (39.3%)	6360/12970 (49.0%)	12,970/12,970 (100%)	6610 (103.9%)
Xin et al. (scRNA-seq) 2016	12,230/39,851 (30.7%)	24,899/39,851 (62.5%)	25,685/39,851 (64.5%)	786 (3.2%)

A reduced amount of features—proteins in this case—is observed when using the default version of HarmonizR. This loss is due to the existence of multiple single-protein matrices, which are not considerable for the underlying adjustment algorithms. The newly implemented functionality resolves this issue and for the largest TMT dataset from Nusinow et al., we were able to increase the amount of rescued features by 6610 in comparison to the old HarmonizR version (103.9% increase) and by 7872 compared to raw ComBat/limma (123.8% increase).

To underline the importance of this additional retention of features, a downstream gene ontology enrichment analysis (GOEA) has been performed exemplarily on the dataset from Krug et al., which is a technique to identify biological processes or molecular functions that are overrepresented in a given set of interest with respect to a universe set; usually the entirety of features (proteins/genes) within the study. Here, the newly rescued proteins are provided as the set of interest. The universe set encompasses the entire list of protein IDs found within the original dataset. The interest set was comprised of 2536 proteins and the universe set of 11,585. The slightly lower numbers than would be expected stem from certain protein IDs not having associated GO annotations or could not be matched to gene symbols. The results of the GOEA is shown in Table 6. The values on the left of the total set counts indicate, how many of these proteins are associated with the respective found GO description.

**Table 6:** Gene ontology enrichment analysis for the newly rescued features/proteins for the dataset from Krug et al., showing the five most significant results

Description	GeneRatio	BgRatio	p-value
Anion transmembrane transport	57/2536	145/11,585	0.0000014
Cell-cell adhesion via plasma-membrane adhesion molecules	63/2536	169/11,585	0.0000034
Axoneme assembly	24/2536	49/11,585	0.0000253
Cilium-dependent cell motility	31/2536	71/11,585	0.0000327
Adenylate cyclase-modulating G protein coupled receptor signaling pathway	32/2536	80/11,585	0.0001880

The impact of the three sorting approaches on blocking and unique feature handling as well as the amount of data loss due to blocking is detailed in Online Appendix Table 1. Their impact in terms of preserved numerical values in the data matrix as well as proteins in the output matrix can be derived from the table. Jaccard-based sorting performing best at a reduction of lost data of ca. 10% for a block parameter of 2 and another ca. 15% if blocking is applied more aggressively (block parameter 4).

Notably, sorting does not meaningfully impact the adjustment quality as shown in Table 7.

**Table 7:** Comparison of the Average Silhouette Widths (ASWs) when using the biological labels for the dataset from Petralia et al. All three sorting approaches are shown alongside no sorting for both block = 2 and block = 4, respectively

Sortings	Petralia et al. (block = 2)	Petralia et al. (block = 4)
No sort	0.086	0.088
Sparsity sort	0.085	0.087
Seriation sort	0.086	0.087
Jaccard sort	0.084	0.086

Blocking is optional and mainly recommended for big datasets or for rapid preliminary analyses. This allows a better tailoring to the users’ needs, be it maximal data preservation or higher runtime efficiency. Whether to sort at all and the choice of the sorting algorithm is highly dependent on the given dataset.

## Discussion

Blocking decreases runtime significantly and thus opens up the use of HarmonizR to even bigger omics datasets such as single cell proteomic datasets as well as single-cell RNA seq data that are known to have a big amount of technical zeros, which are effectively missing values [2].

Besides, the rapid growth of data bank entries [26] poses more and more run time challenges to data integration routines, which motivates and justifies the present HarmonizR version, a computationally even more efficient algorithmic solution to the batch effect reduction step. The blocking trades a slight data loss during batch effect reduction for computational efficiency. As mentioned before, computational efficiency is often aligned with certain drawbacks in terms of accuracy of the algorithm in question, yet, the here proposed approach does yield a very tolerable trade as long as parameters are chosen fittingly to dataset size and within reason.

Additionally, to some extent, any present data loss can be mitigated by the use of the sorting parameter and the removal of unique combinations. The latter, as one of the new additions, will yield a greatly reduced data loss in most cases and works particularly well in tandem with the blocking approach as this new version of HarmonizR can simultaneously successfully adjust the input data in less time and still yield a higher number of remaining features within the batch effect corrected output data.

Generally, this new HarmonizR update can be applied to any other big data with internal biases.

## Conclusions

The apparent trend of growing data availability increases the necessity for efficient methods—as implemented here for HarmonizR—that are suited for large, integrated datasets. For this, a blocking strategy was conceived to reduce the amount of produced sub-matrices within HarmonizR’s dissection approach, which significantly reduces runtime. Output quality is comparable to default HarmonizR for reasonable blocking sizes 2 and 4.

To overcome ComBat’s and limma’s limitation not to work with unique feature combinations, the novel unique removal strategy uses minimalistic cropping to effectively remove a feature’s uniqueness and enable it for subsequent adjustment. With this new approach we observe drastic improvements in data rescue for both blocking and non-blocking approaches.

Our results show a block size of 2 to yield a good compromise between performance and accuracy.

## Supplementary Information

Supplementary file 1.

## Data Availability

The datasets used and/or analyzed during the current study are available from the corresponding author on reasonable request. Three publicly available TMT datasets have been used in this work. The first dataset was published and provided in 2020 by Krug et al. (153 samples distributed over 17 batches/13,793 features, 2932 of them unique/500,696 missing values, making up 23.73% of the dataset) [17]. The second dataset was published in 2020 by Petralia et al. (249 samples distributed over 23 batches/9155 features, 3938 of them unique/657,917 missing values, making up 28.86% of the dataset) [18]. The third dataset was published in 2020 by Nusinow et al. (420 samples distributed over 42 batches/12,970 features, 6610 of them unique/1,555,521 missing values, making up 28.56% of the dataset) [19]. The used single cell dataset was published by Xin et al. in 2016 (651 samples distributed over 12 batches/39,851 features, 786 of them unique/22,032,318 missing values, making up 84.93% of the dataset) [20]. The data accession numbers can be derived from the original publications referenced above. The HarmonizR algorithm has been used as a basis for this update [12]. It is fully implemented in the R programming language. The majority of the figures has been created using Python’s matplotlib and seaborn libraries for data plotting and visualization. The introduced block parameter is optional and can be set to any number between 1 and the number of batches within the dataset. The passed value determines the number of batches to be blocked together during HarmonizR execution. Blocking is turned off by default. The introduced sort parameter is optional and can be set to either “sparsity”, “seriation” or “jaccard”. The chosen sorting method is then used to rearrange the batches prior to blocking. This usually leads to lower data loss during blocking. The data will be re-sorted to its default order prior to the HarmonizR adjustment. Sorting is turned off by default. The unique removal approach has its own parameter called “ur”, which can be set by the user. Unique removal is turned on by default and it is advised against switching it off. Yet, the option to do so is provided due to result reproducibility reasons. The new, updated and documented HarmonizR package is available on GitHub: https://github.com/HSU-HPC/HarmonizR. The here shown update has been compared to the original HarmonizR version [12], which is available on GitHub within the same repository as a different release (tag: HarmonizR_Nature_Communications). The here introduced HarmonizR version has been reviewed for and published on Bioconductor (https://www.bioconductor.org/packages/release/bioc/html/HarmonizR.html).
