# Supplementary material for: HarmonizR: blocking and singular feature data adjustment improve runtime efficiency and data preservation
Source: BMC Bioinformatics. 2025 Feb 11;26:47. doi: 10.1186/s12859-025-06073-9 (PMC11817103; doi:10.1186/s12859-025-06073-9)
Supplement: Supplementary file 1 [file 12859_2025_6073_MOESM1_ESM.pdf]

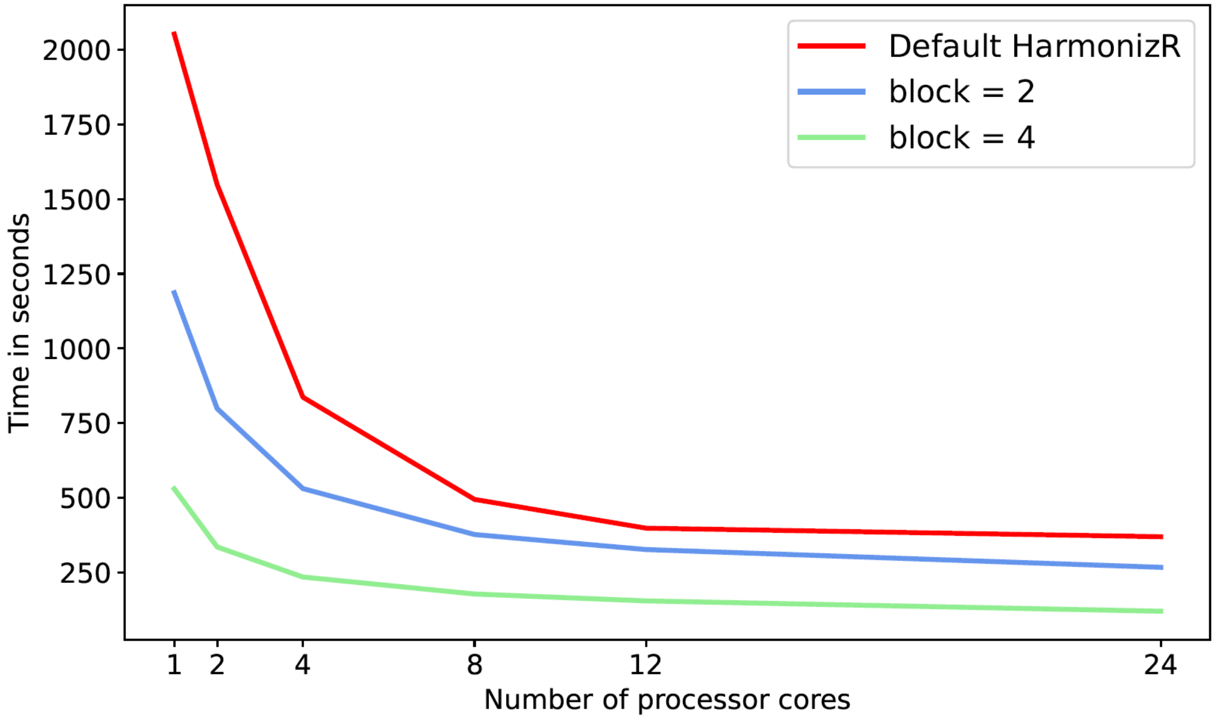

Figure 1: Runtime of HarmonizR on an artificially created dataset with dimensions 25000 (features) times 1000 (samples). Values are normally distributed and missing values (20 percent) were introduced missing-not-at-random. Runs have been performed using no blocking as well as blocking parameters 2 and 4 using 1, 2, 4, 8, 12 and 24 precessor cores, respectively. Computed on an Intel Xeon Gold 6226, 2.70 GHz, 2 × 12 compute cores, 96 GB RAM. Measured with 5 times repetition with the mean shown.

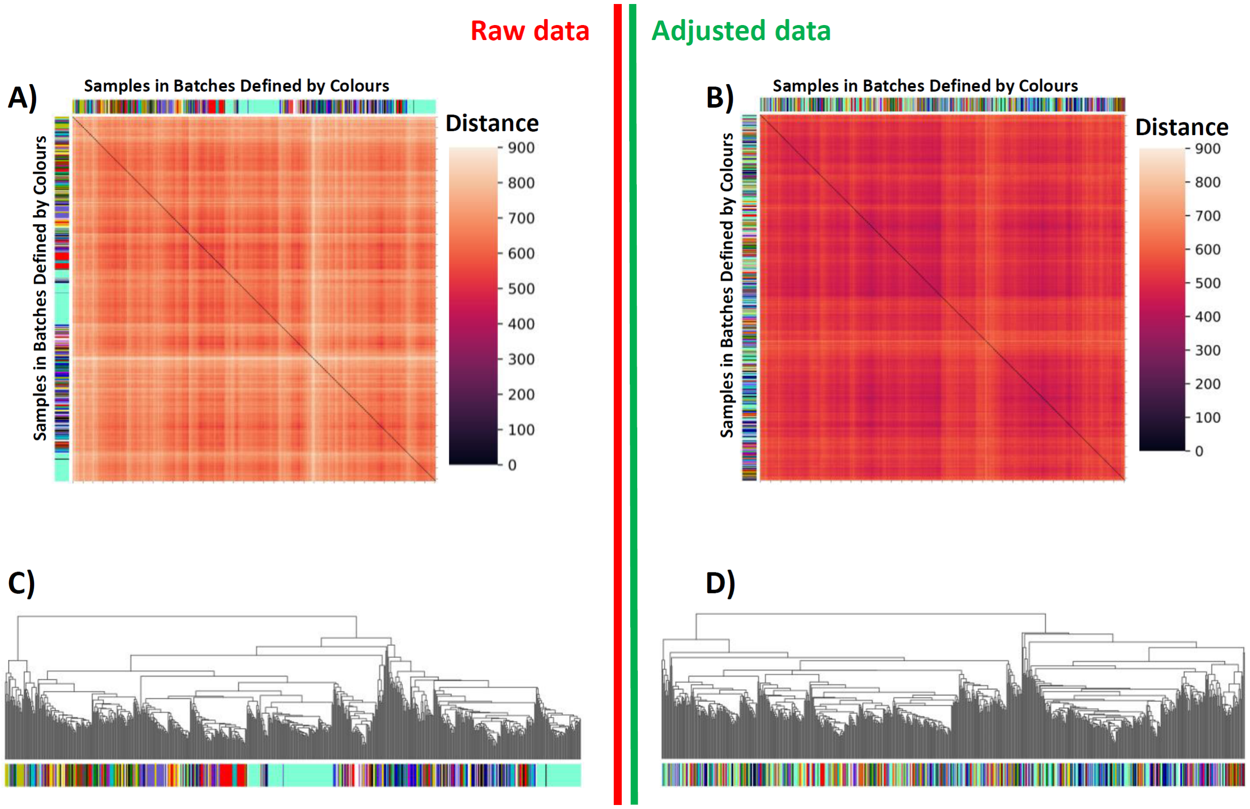

Figure 2: Tests for HarmonizR on a Pancreas single-cell-RNA-seq dataset from Xin et al. from 2016. A total of 651 samples are found within 12 batches. The raw data is depicted on the left. The adjusted data, showing a reduced batch effect, is depicted on the right. Adjustments were done using ComBat and no prior sorting. **A)** Heatmap representation of the distance matrices (all samples vs. all samples) for the unadjusted dataset. **B)** Heatmap representation of the distance matrices (all samples vs. all samples) after batch effect reduction. **C)** Dendrogram representation of clustering by batches for the unadjusted dataset. **D)** Dendrogram representation of clustering by batches after batch effect reduction.

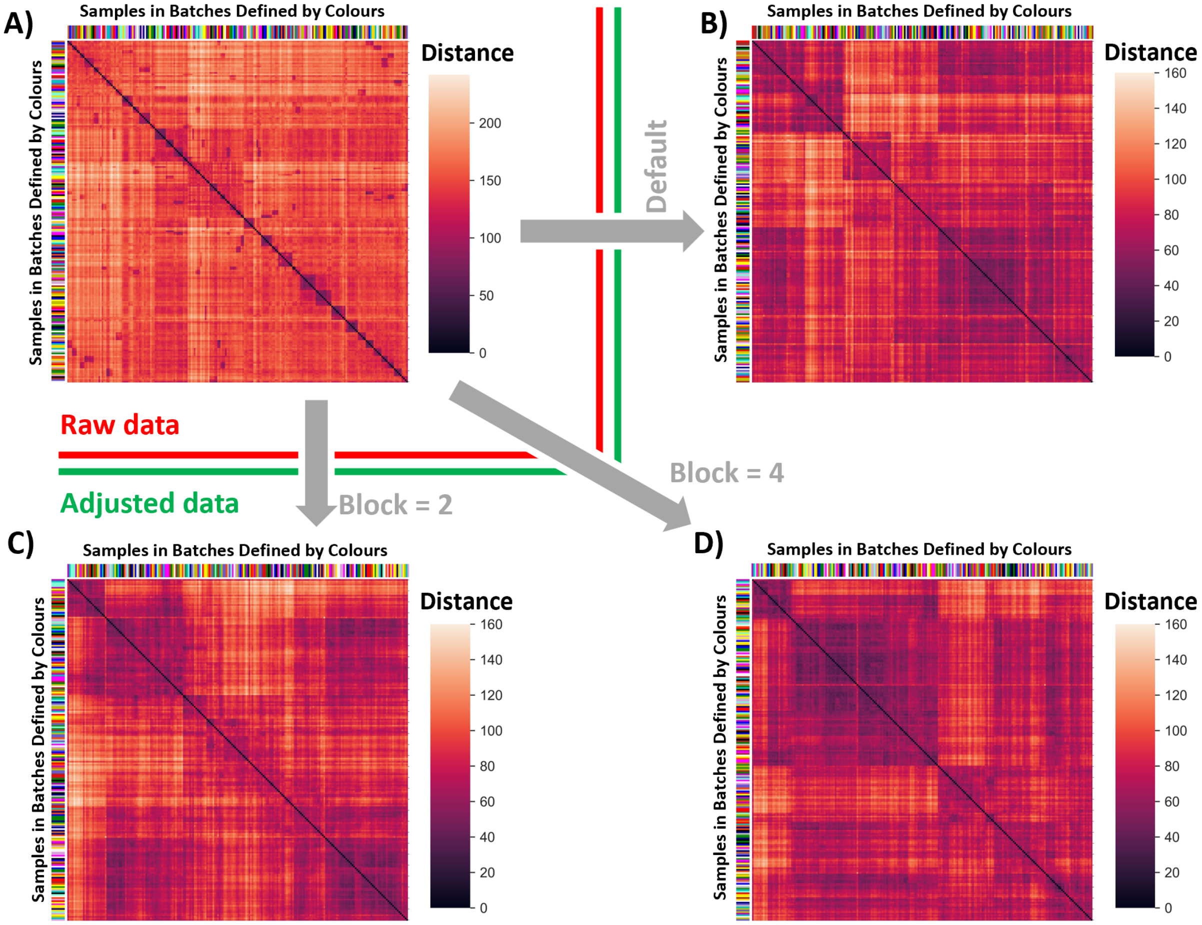

Figure 3: Heatmap representation of the distance matrices calculated from the dataset from Petralia et al. (all samples vs. all samples); raw, adjusted by the default version of HarmonizR and lastly adjusted using the blocking approach. Next to the heatmaps, the respective legend is shown, defining the meaning of the shading. Color labels for samples indicate assignment to a certain batch. All adjustments were done using ComBat and without prior sorting. **A)** The raw data. **B)** The adjusted data from the default version of HarmonizR without blocking. **C)** The data arising from the blocking approach with the block parameter equal to 2. **D)** The data arising from the blocking approach with the block parameter equal to 4.

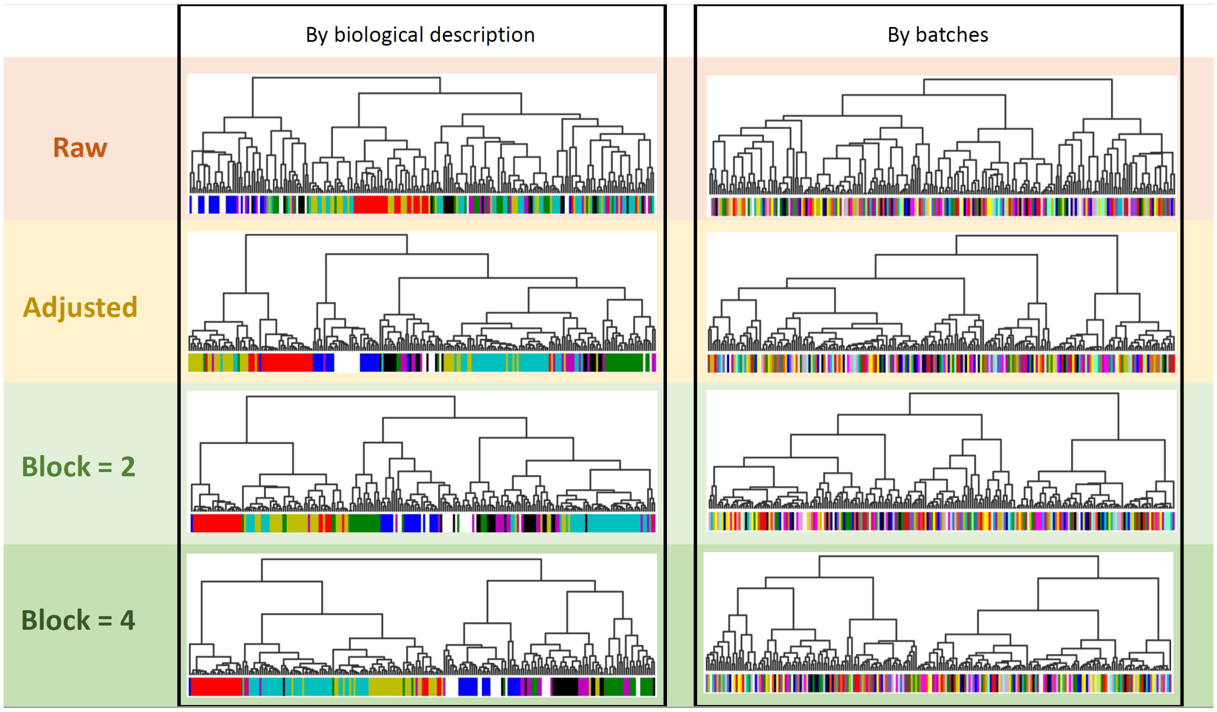

Figure 4: Dendrogram representation of clustering by batches and by biological description. This is done for Petralia et al. with the raw dataset, the adjusted dataset (using the default version of HarmonizR) and the adjusted dataset under the usage of blocking. block = 2 means always blocking 2 batches together if possible. block = 4 means always blocking 4 batches together, respectively. Leaves of the same color indicate the same affiliation of these samples in terms of either biology (left) or the batch they were measured in (right). All adjustments were done using ComBat as sub-matrix batch effect adjustment method without prior sorting.

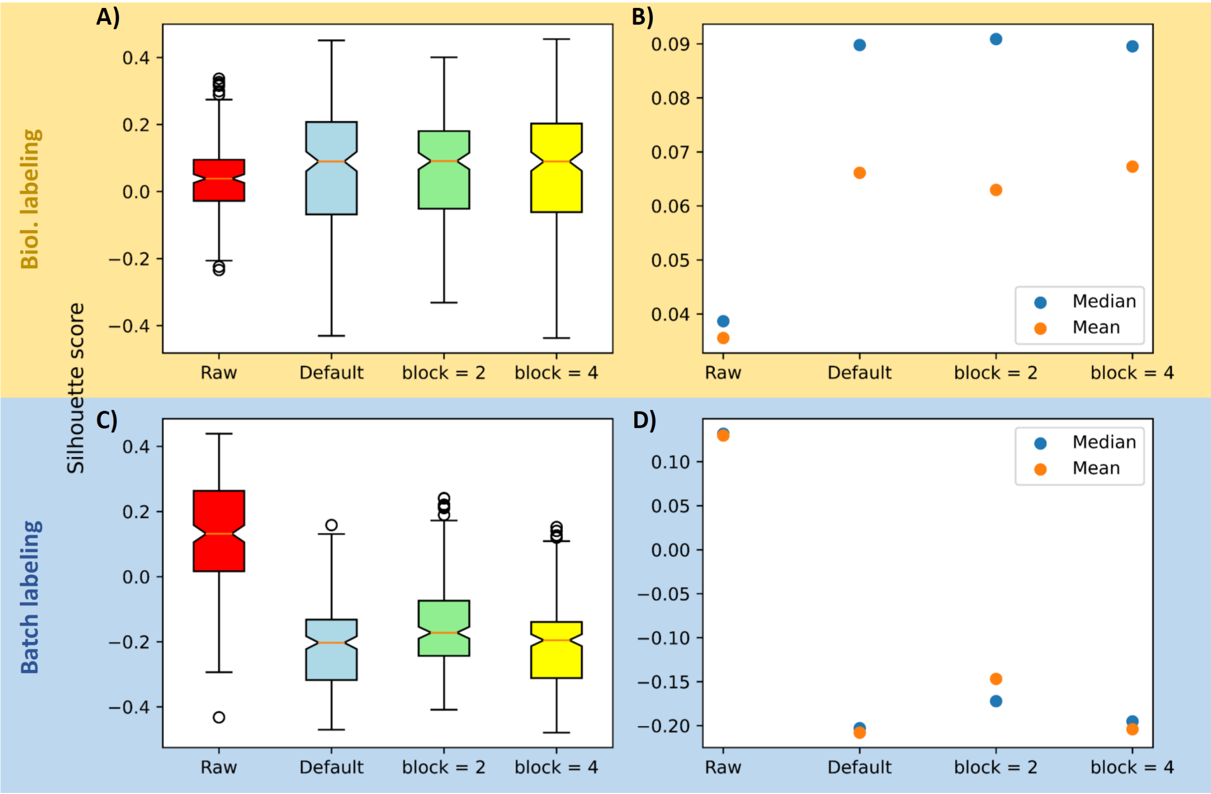

Figure 5: Silhouette score calculated for the dataset from Petralia et al.. The given granularity is shown on the x-axis. The silhouette score, shown on the y-axis, lies between -1 and 1. 0 means randomly distributed, 1 means perfect clusters (all data points on top of each other within a cluster). -1 describes the opposite of 1. All adjustments were done using ComBat in its default mode as sub-matrix batch effect adjustment method without prior sorting. **A)** Boxplot depiction of the silhouette score using biological labeling. **B)** The median (blue) and the mean (orange) of the boxplots directly to the left (biol. labeling). **C)** Boxplot depiction of the silhouette score using labeling by batches. **D)** The median (blue) and the mean (orange) of the boxplots directly to the left (batch labeling).
In boxplots, 50 % of the data points are inside the box (Q1 (Quartile 1) being the lower bound of the box (25 %), Q3 being the upper bound of the box (75 %)). Whiskers show all values beyond the box without outliers. Outliers were defined as Q3+ 1.5 * IQR (Interquartile range) (upper outlier) and Q1-1.5 * IQR (lower outlier). IQR being Q1–Q3.

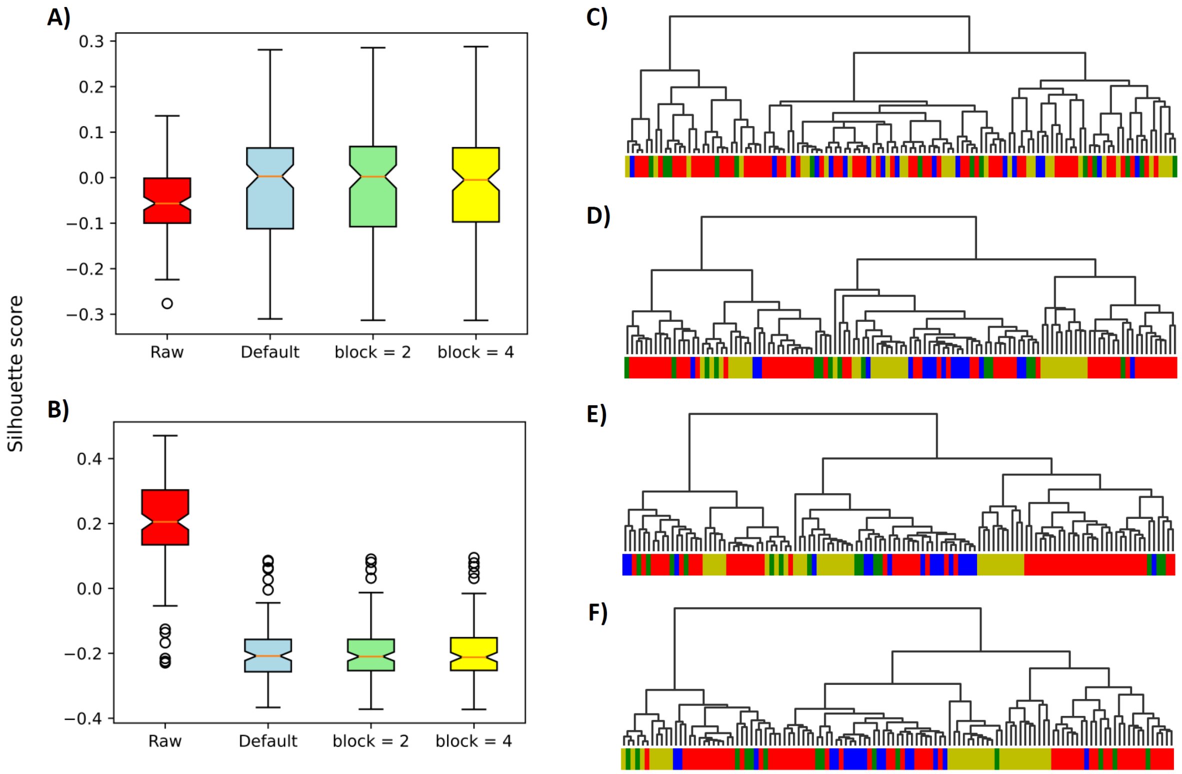

Figure 6: Silhouette score and clustering performed on the dataset from Krug et al. with newly introduced missing values at random (increasing the amount from 23.73 % to 50.39 %). All adjustments were done using ComBat as sub-matrix batch effect adjustment method without prior sorting. **A)** Boxplot depiction of the silhouette score using biological labeling. **B)** Boxplot depiction of the silhouette score using batch labeling. **C)** Clustering of the raw data with biological labels. **D)** Clustering of the adjusted data without blocking (using biological labels). **E)** Clustering of the adjusted data with the blocking parameter set to 2 (using biological labels). **F)** Clustering of the adjusted data with the blocking parameter set to 4 (using biological labels).
In boxplots, 50 % of the data points are inside the box (Q1 (Quartile 1) being the lower bound of the box (25 %), Q3 being the upper bound of the box (75 %)). Whiskers show all values beyond the box without outliers. Outliers were defined as Q3+ 1.5 * IQR (Interquartile range) (upper outlier) and Q1-1.5 * IQR (lower outlier). IQR being Q1–Q3.

Table 1: Overview over lost numerical values within the data matrix as well as present proteins (genes in case of the dataset from Xin et al.) in the output matrix during blocking. All three sorting strategies are shown in comparison. Measurements were done for no blocking, block = 2 and block = 4. No sorting was applied when blocking was not used (Setting = Normal). For lost numerical values, a lower number / percentage is desirable, for proteins within the output, a higher number / percentage is desirable. Rescuing of features with unique combinations was active for all runs.

Dataset	Setting	Sparsity sort<br>→ Num. values lost<br>→ Proteins in output	Seriation sort<br>→ Num. values lost<br>→ Proteins in output	Jaccard sort<br>→ Num. values lost<br>→ Proteins in output
Krug et al. 2020	Normal	→ 6 (≈ 0 %)<br>→ 13740/13793 (99.6 %)		
	block = 2	→ 149346 (9.3 %)<br>→ 12010/13793 (87.1 %)	→ 144684 (9.0 %)<br>→ 12107/13793 (87.8 %)	→ 131732 (8.2 %)<br>→ 12505/13793 (90.7 %)
	block = 4	→ 267138 (16.6 %)<br>→ 10562/13793 (76.6 %)	→ 266032 (16.5 %)<br>→ 10591/13793 (76.8 %)	→ 260256 (16.2 %)<br>→ 11583/13793 (84.0 %)
Petralia et al. 2020	Normal	→ 1584 (0.1 %)<br>→ 9154/9155 (≈ 100 %)		
	block = 2	→ 203902 (12.6 %)<br>→ 7959/9155 (86.9 %)	→ 202033 (12.5 %)<br>→ 8176/9155 (89.3 %)	→ 185869 (11.5 %)<br>→ 8191/9155 (89.5 %)
	block = 4	→ 370367 (22.8 %)<br>→ 6390/9155 (69.8 %)	→ 363542 (22.4 %)<br>→ 6587/9155 (72.0 %)	→ 372048 (22.9 %)<br>→ 6374/9155 (69.6 %)
Nusinow et al. 2020	Normal	→ 0 (0 %)<br>→ 12970/12970 (100 %)		
	block = 2	→ 468856 (12.1 %)<br>→ 11435/12970 (88.2 %)	→ 429305 (11.0 %)<br>→ 11576/12970 (89.3 %)	→ 393338 (10.1 %)<br>→ 11729/12970 (90.4 %)
	block = 4	→ 814401 (20.9 %)<br>→ 9542/12970 (73.6 %)	→ 751778 (19.3 %)<br>→ 9905/12970 (76.4 %)	→ 780213 (20.1 %)<br>→ 9719/12970 (74.9 %)
Xin et al. 2016	Normal	→ 47011 (1.2 %)<br>→ 25685/39851 (64.5 %)		
	block = 2	→ 128526 (3.3 %)<br>→ 20833/39851 (52.3 %)	→ 128526 (3.3 %)<br>→ 20833/39851 (52.3 %)	→ 159756 (4.1 %)<br>→ 20354/39851 (51.1 %)
	block = 4	→ 213565 (5.5 %)<br>→ 17258/39851 (43.3 %)	→ 213565 (5.5 %)<br>→ 17258/39851 (43.3 %)	→ 258134 (6.6 %)<br>→ 16508/39851 (41.4 %)
